# Promoting Heme and Phycocyanin Biosynthesis in *Synechocystis* sp. PCC 6803 by Overexpression of Porphyrin Pathway Genes with Genetic Engineering

**DOI:** 10.3390/md21070403

**Published:** 2023-07-13

**Authors:** Kai Cao, Xiaodong Wang, Fengjie Sun, Hao Zhang, Yulin Cui, Yujiao Cao, Qingshou Yao, Xiangyu Zhu, Ting Yao, Meng Wang, Chunxiao Meng, Zhengquan Gao

**Affiliations:** 1 School of Life Sciences and Medicine, Shandong University of Technology, Zibo 255049, China; 17852032808@163.com (K.C.); plamanwxd@outlook.com (X.W.); 2 School of Pharmacy, Binzhou Medical University, Yantai 264003, China; haoge073711@163.com (H.Z.); cyl-lc@163.com (Y.C.); yaoqingshou@126.com (Q.Y.); zhuxyscut@163.com (X.Z.); 21410010896@stumail.sdut.edu.cn (T.Y.); 3 Department of Biological Sciences, School of Science and Technology, Georgia Gwinnett College, Lawrenceville, GA 30043, USA; fsun@ggc.edu; 4 School of Foreign Languages, Shandong University of Technology, Zibo 255090, China; 21514011000@stumail.sdut.edu.cn; 5 Yantai Hongyuan Bio-Fertilizer Co., Ltd., Yantai 264000, China; 15106527617@163.com

**Keywords:** heme, phycocyanin, gene expression, synthetic mechanism, *Synechocystis* sp. PCC 6803, *Synechococcus* elongatus PCC 7942

## Abstract

Due to their unique biochemical and spectroscopic properties, both heme and phycocyanobilin are widely applied in the medical and food industries. *Synechocystis* sp. PCC 6803 contains both heme and phycocyanin, and is capable of synthesizing phycocyanin using heme as a precursor. The aim of this study was to uncover viable metabolic targets in the porphyrin pathway from *Synechocystis* sp. PCC 6803 to promote the accumulation of heme and phycocyanin in the recombinant strains of microalgae. A total of 10 genes related to heme synthesis pathway derived from *Synechococcus elongatus* PCC 7942 and 12 genes related to endogenous heme synthesis were individually overexpressed in strain PCC 6803. The growth rate and pigment content (heme, phycocyanin, chlorophyll a and carotenoids) of 22 recombinant algal strains were characterized. Quantitative real-time PCR technology was used to investigate the molecular mechanisms underlying the changes in physiological indicators in the recombinant algal strains. Among the 22 mutant strains, the mutant overexpressing the haemoglobin gene (*glbN*) of strain PCC 6803 had the highest heme content, which was 2.5 times higher than the wild type; the mutant overexpressing the gene of strain PCC 7942 (*hemF*) had the highest phycocyanin content, which was 4.57 times higher than the wild type. Overall, the results suggest that genes in the porphyrin pathway could significantly affect the heme and phycocyanin content in strain PCC 6803. Our study provides novel crucial targets for promoting the accumulation of heme and phycocyanin in cyanobacteria.

## 1. Introduction

Heme (C_34_H_33_FeN_4_O_4_) is a small molecule containing protoporphyrin and ligand divalent iron ions. At the same time, it is also an important cofactor involved in a variety of biological processes, e.g., oxygen transport and storage, electron transfer, drug and steroid metabolism, signal transduction, and microRNA processing [1]. However, excess free heme is highly toxic, promoting oxidative stress and lipid peroxidation, ultimately leading to membrane damage and apoptosis. Both biliverdin and bilirubin derived from the degradation of heme maintain the intracellular redox balance [2]. Heme also acts as a pro-oxidant, involved in the production of reactive oxygen species (ROS) required for growth and differentiation processes [3]. And that is also a precursor for phycocyanobilin (C_33_H_38_N_4_O_6_), which is generally bound to proteins and is mainly found in cyanobacteria as phycocyanin [4], involved in efficient energy transfer.

Heme determines the flavour, colour, and iron content of meat. Studies have shown that the addition of that to plant-based meat can significantly enhance the flavour and appearance of artificial meat [5]. The plant-based meat market is expected to increase from $4.6 billion in 2018 to $85 billion by 2030 [6]. However, the saturation of the plant-based meat market is extremely low, mainly due to the limited sources of heme, which fall far short of demand. As a new and highly effective iron supplement, it could be used to treat iron deficiency anaemia [7]. Both heme and phycocyanin are excellent natural food colouring agents that could replace traditional colouring agents such as nitrite and synthetic colours to ensure the food safety and reduce carcinogenic factors [8]. Furthermore, due to its unique photosensitive properties and medicinal values, heme could also be used in the diagnosis of diseases and in the preparation of potent anti-cancer drugs. Phycocyanin has shown significant anti-cancer, anti-diabetic, anti-inflammatory, antioxidant, and immunomodulatory functions [9,10], with high promises to be a novel and effective drug for the treatments of many diseases such as COVID-19 complications, atherosclerosis, multiple sclerosis, and ischaemic stroke [11,12]. Phycocyanin has also been revealed to have protective effects on brain, liver, kidney, intestine, and reproductive system [13,14,15,16]. Moreover, the in vitro experiments demonstrated that phycocyanin peptide is capable of reducing the oxidative and inflammatory cellular damages, thereby alleviating the lung fibrosis [17].

Currently, there are three main sources of heme on the market, each with various productive and biological disadvantages. The first source is fresh pig blood. However, there are many limitations in the collection, transportation, and storage of raw materials, seriously polluting the environment. The second source is extracted from plant materials such as soybean roots. However, the long seasonal production cycle of soybeans is not conducive to large-scale production of heme. Third, heme is produced by chemical synthesis. Although heme can be also chemically synthesized, complicated synthesis procedures and low yield makes the heme expensive [18]. In addition, compared to chemically synthesized heme, heme of biological origin is easily absorbed and utilized by human cells without any side effects [19]. Therefore, the development of microbe-based heme production is a potential solution. Although *Spirulina* is generally a natural source of commercially produced phycocyanin, it is susceptible to contamination caused by other cyanobacteria, which are capable of producing toxins harmful to liver and nerves. For example, *Spirulina* biomass is revealed to have high metal accumulation capacity [20], reducing the safety of phycocyanin. *Synechocystis* sp. PCC 6803 is a unicellular cyanobacterium that is both autotrophic and heterotrophic. *Synechocystis* sp. PCC 6803 can be cultured in freshwater or seawater to produce high value-added products [21,22]. Compared with other cyanobacterial taxa, strain PCC 6803 has shown several unique advantages in its application in genetic modification, i.e., (i) cells of strain PCC 6803 are fast growing, highly adaptable, non-toxic, and non-polluting; (ii) the genetic background of strain PCC 6803 has been extensively investigated; and (iii) cells of strain PCC 6803 are natural receptors [23].

**Figure 1:**
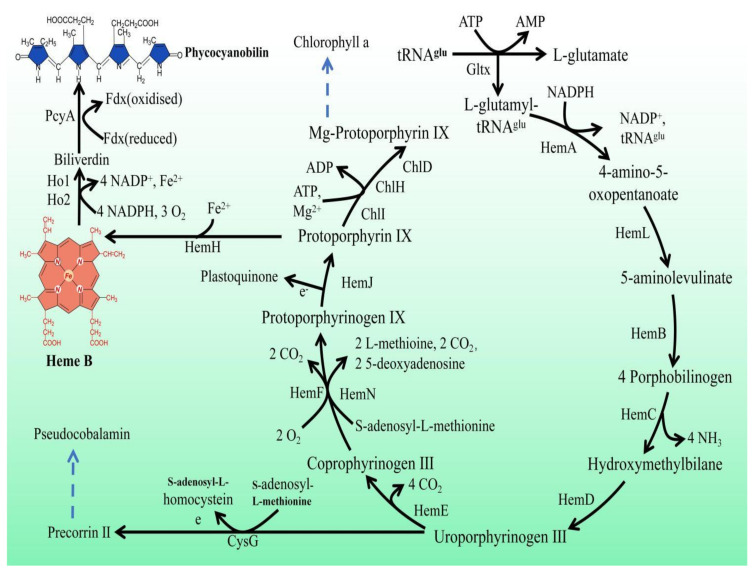
Porphyrin metabolic pathway in *Synechocystis* sp. PCC 6803. The blue dashed arrows represent multi-step enzymatic reactions. Gltx, glutamyl-tRNA synthetase; HemA, glutamyl-tRNA reductase; HemL,glutamate-1-semialdehyde 2,1-aminomutase; HemB, porphobilinogen synthase; HemC, hydroxymethylbilane synthase; HemD, uroporphyrin-III synthase; HemE, uroporphyrinogen decarboxylase; HemF, coproporphyrinogen III oxidase; HemN, oxygen-independent coproporphyrinogen III oxidase; HemJ, protoporphyrinogen IX oxidase; HemH, protoporphyrin/coproporphyrin ferrochelatase; Ho1/Ho2, heme oxygenase; PcyA, phycocyanobilin: ferredoxin oxidoreductase; CysG, uroporphyrin-III C-methyltransferase; ChlI, magnesium chelatase subunit I; ChlH, magnesium chelatase subunit H; ChlD, magnesium chelatase subunit D.

In *Synechocystis* sp. PCC 6803, heme is a precursor of phycocyanin and is synthesized by L-glutamic acid and tRNA^Glu^ in ten enzymatic steps (Figure 1). First, the reaction of L-glutamyl and tRNA^Glu^ is catalyzed by glutamyl-tRNA synthetase (Gltx) to produce L-glutamyl-tRNA^Glu^. Then, L-glutamate-1-semialdehyde is produced via glutamyl-tRNA reductase (HemA) and 5-aminolevulinic acid (5-ALA) is generated via glutamate-1-semialdehyde-2,1-aminomutase (HemL). 5-ALA is an important precursor of heme synthesis, which is sequentially transformed into protoporphyrinogen IX by a group of enzymes, including porphobilinogen synthase (HemB), porphobilinogen deaminase (HemC), uroporphyrin III synthase (HemD), uroporphyrinogen decarboxylase (HemE), coproporphyrinogen oxidases (HemF and HemN), and protoporphyrinogen Ⅸ oxidase (HemJ). Ultimately, heme can be bound to haemoglobin (glbn) and stored in algal cells or be catalyzed by the heme oxygenases (Ho1 and Ho2) to generate biliverdin, which is further catalyzed by phycocyanobilin: ferredoxin oxidoreductase (PcyA) to produce phycocyanin.

In this study, a total of 22 porphyrin pathway genes derived from *Synechocystis* sp. PCC 6803 and *Synechococcus elongatus* PCC 7942 were individually overexpressed in strain PCC 6803, respectively. The goal of this study was to uncover viable metabolic targets in the porphyrin pathway from *Synechocystis* sp. PCC 6803 to promote the accumulation of heme and phycocyanin in the recombinant strains of microalage. The growth rate and the contents of high value-added pigments (heme, phycocyanin, chlorophyll a, and carotenoids) were characterized in the mutant and wild type (WT) of PCC 6803. A quantitative real-time PCR (qRT-PCR) technology was used to study the changes in heme and phycocyanin synthesis gene expression and their competing pathways in the mutant strains. To our knowledge, this is the first exploration of metabolic targets in the porphyrin synthesis pathway to promote heme and phycocyanin accumulation in microalgae.

## 2. Results

### 2.1. Overexpression of Heme Synthesis Genes in Synechocystis sp. PCC 6803

**Figure 2:**
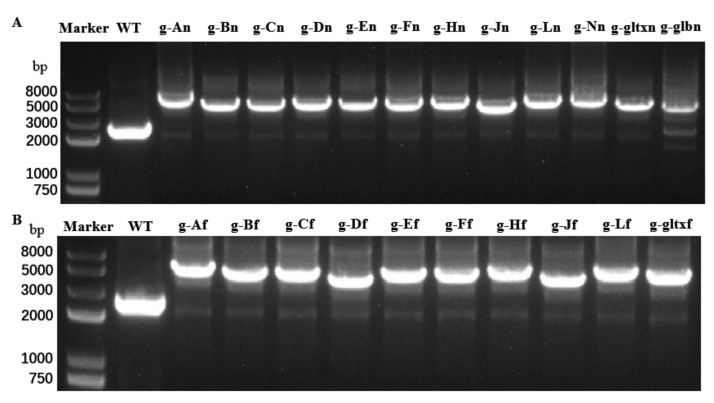
PCR amplifications of (**A**) 12 endogenous heme synthesis gene expression cassettes of *Synechocystis* sp. PCC6803 and (**B**) 10 exogenous heme synthesis gene expression cassettes of *Synechococcus* elongatus PCC 7942 based on the genomes of the wild type (WT) and mutant strains of *Synechocystis* sp. PCC 6803.

To isolate and expand candidate overexpressing colonies to generate clonal populations, individual resistant colonies on the screening plate were selected and delineated on a new screening plate. This process was repeated for five generations with gradually increased concentrations of antibiotics in the plates. During the fifth generation, the genomic DNA of the mutant strain was extracted and used as template for PCR analysis. The 12 mutant strains with overexpression of heme synthesis genes derived from strain PCC 6803 were designated as g-An (overexpressing strain PCC 6803 *hemA* gene), g-Bn, g-Cn, g-Dn, g-En, g-Fn, g-Hn, g-Jn, g-Ln, g-Nn, g-glbn, and g-gltxn, respectively. The 10 mutant strains with overexpression of heme synthesis genes derived from strain PCC 7942 were named g-Af (overexpressing strain PCC 7942 *hemA* gene), g-Bf, g-Cf, g-Df, g-Ef, g-Fn, g-Hf, g-Jf, g-Lf, and g-gltxf, respectively. The entire inserted DNA fragments were PCR-amplified with the products electrophoresed (Figure 2). The results of electropherogram and DNA sequencing showed that all 22 gene expression cassettes were successfully inserted into the mutant strains, respectively.

### 2.2. Western Blot Analysis

**Figure 3:**
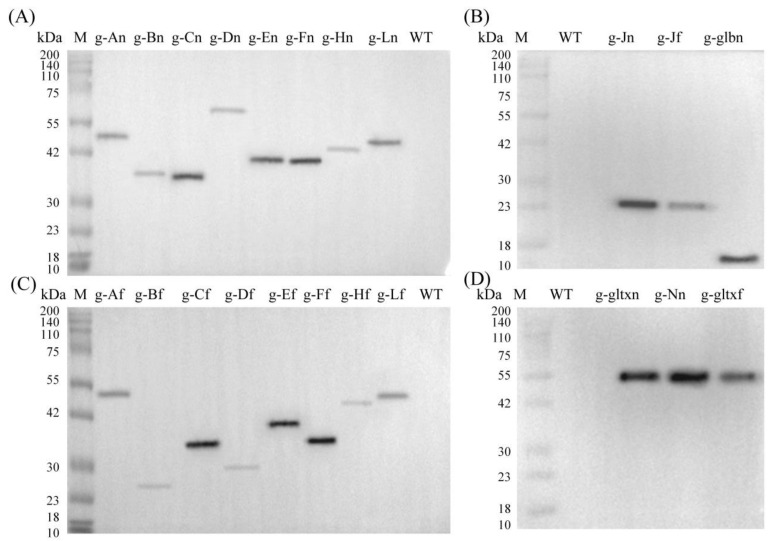
Western blot analyses of the WT and mutant strains of *Synechocystis* sp. PCC 6803 based on (**A**,**B**) 12 heme synthesis genes of *Synechocystis* sp. PCC 6803 and (**C**,**D**) 10 heme synthesis genes of *Synechococcus elongatus* PCC 7942.

Based on the Western blot analysis, the molecular weights of the target proteins overexpressed in the mutant strains of PCC 6803 and their sources were given in Table S6. The results showed that the bands with expected sizes were obtained in all mutant strains but not in the WT, indicating that the mutant strains were successfully constructed with the target genes expressed (Figure 3).

### 2.3. Growth of WT and Mutant Strains of Synechocystis sp. PCC 6803

**Figure 4:**
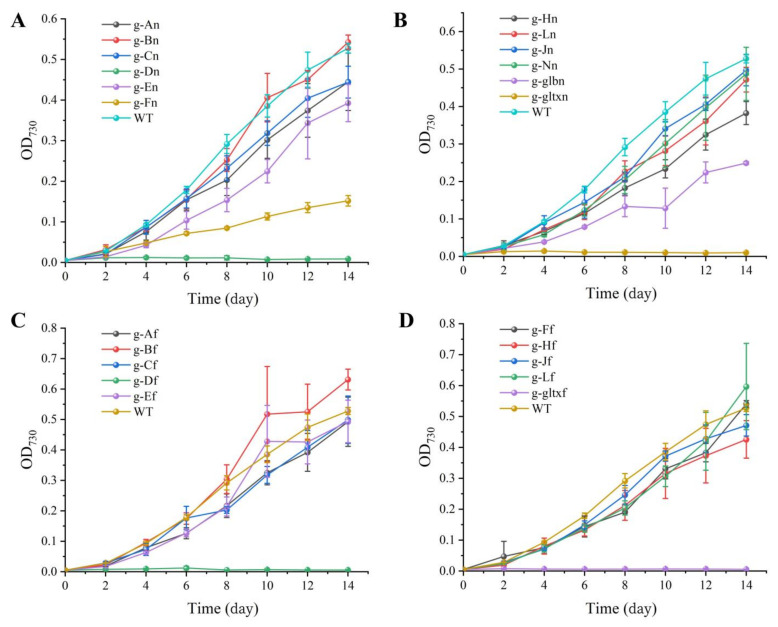
Growth curves of WT and mutant strains of *Synechocystis* sp. PCC 6803. (**A**) Wild-type and mutants overexpressing endogenous genes of PCC 6803. (**B**) Mutant strains overexpressing endogenous genes of PCC 6803. The mutant strains g-An, g-Bn, g-Cn, g-Dn, g-En, g-Fn, g-Hn, g-Jn, g-Ln, g-Nn, g-glbn, and g-gltxn are overexpressed with the endogenous genes of *HemA*, *HemB*, *HemC*, *HemD*, *HemE*, *HemF*, *HemH*, *HemJ*, *HemL*, *HemN*, *glbN,* and *gltX*, respectively, in PCC 6803. (**C**) Wild-type and mutant strains with overexpression of genes of *Synechococcus elongatus* PCC 7942. (**D**) Mutant strains with overexpression of genes of PCC 7942. The mutant strains g-Af, g-Bf, g-Cf, g-Df, g-Ef, g-Ff, g-Hf, g-Jf, g-Lf, and g-gltxf are overexpressed with genes *HemA*, *HemB*, *HemC*, *HemD*, *HemE*, *HemF*, *HemH*, *HemJ*, *HemL*, and *gltX*, respectively, in PCC 7942.

The growth curves (Figure 4) showed that g-Bn, g-Bf, g-Lf, and g-Ff grew slower than WT in the early stage, and the growth curves in the later stage were close to WT and finally exceeded its growth rate. Among them, the g-Bf mutant strain had approximately the same growth curve as WT in the first eight days, but surpassed WT on day 8. The mutant strains g-Bn, g-Lf, and g-Ff had slower growth than WT in the early stage, but surpassed WT on day 14 by OD_730_. The other strains had slower growth than WT, among which were g-An, g-Cn, g-Jn, g-Ln, g-Nn, g-Af, g-Cf. The g-Fn and g-glbn mutants were the two slowest growing strains. g-Dn, g-Df, g-gltxn and g-gltxf are strains that used the inducible form of promoter PnrsB, and the addition of inducer (5 μM Ni^2+^ and 6 μM Co^2+^) inhibited the growth of the strains, which was slower than WT. gltxn and g-gltxf biomass decreased on the fourth day, and g-Df started to decrease on the sixth day and eventually died.

### 2.4. Contents of Heme in WT and Mutant Strains of Synechocystis sp. PCC 6803

**Figure 5:**
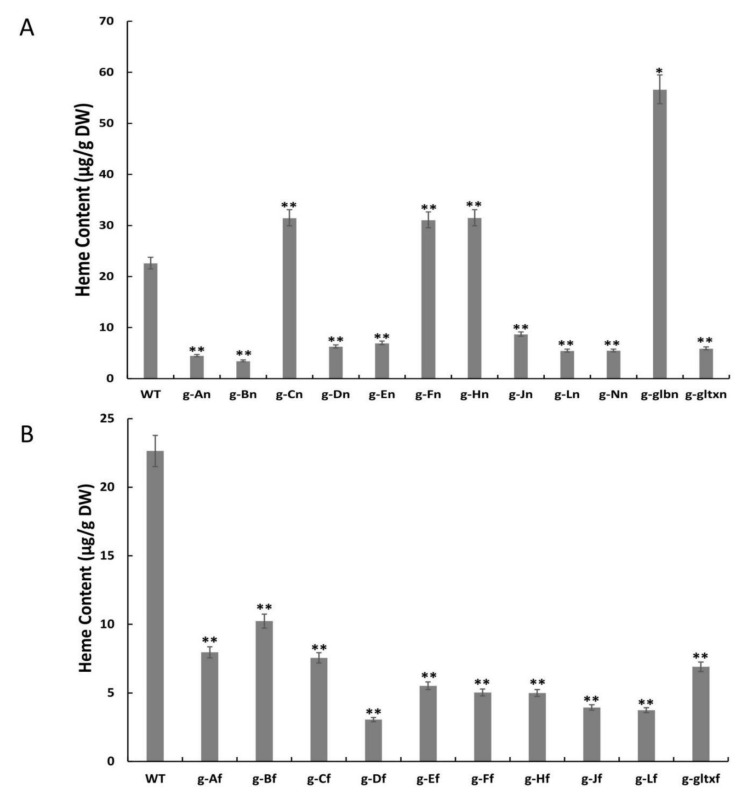
Contents of heme in WT and mutant strains of *Synechocystis* sp. PCC 6803 based on the overexpression of (**A**) 12 heme synthesis genes from *Synechocystis* sp. PCC 6803 and (**B**) 10 heme synthesis genes from *Synechococcus elongatus* PCC 7942. The symbol “*” indicates a significant difference compared to WT (*p* < 0.05), and the symbol “**” indicates a highly significant difference compared to WT (*p* < 0.01).

The contents of heme in the microalgal strains were measured using the fluorometric method (Figure 5). The results showed that the contents of heme were significantly increased in four mutant strains, including g-glbn [56.65 μg/g DW (dry weight)], g-Hn (31.53 μg/g DW), g-Cn (31.51 μg/g DW), and g-Fn (31.09 μg/g DW), in comparison with the WT (22.64 μg/g DW). Among them, the g-glbn mutant strain had the highest heme content, reaching 2.5 times that of the WT. All other overexpression strains showed significantly lower heme contents than that of the WT, with the strain g-Df showing the lowest heme content of 3.05 μg/g DW.

### 2.5. Contents of Chlorophyll a in WT and Mutant Strains of Synechocystis sp. PCC 6803

**Figure 6:**
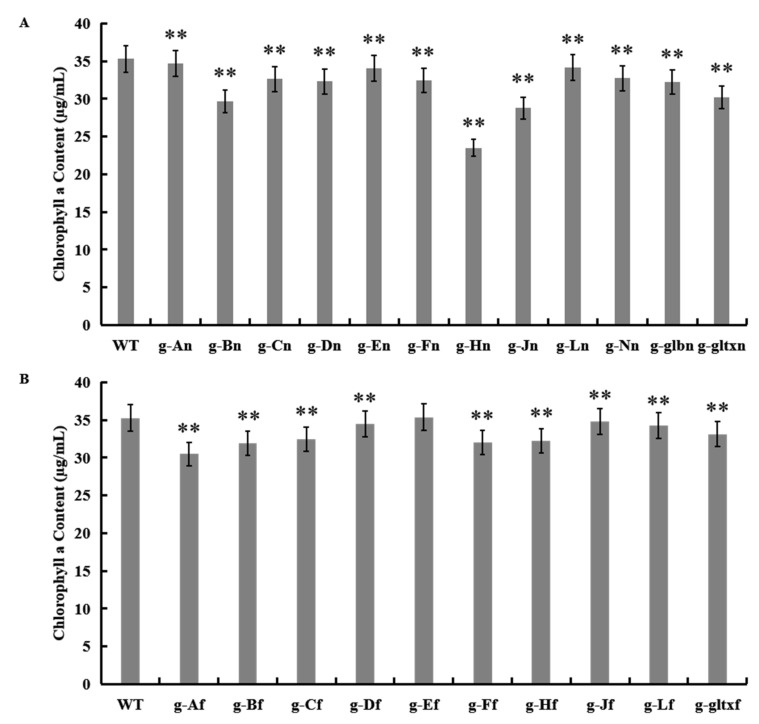
Contents of chlorophyll a in WT and mutant strains of *Synechocystis* sp. PCC 6803 based on the overexpression of (**A**) 12 heme synthesis genes from *Synechocystis* sp. PCC 6803 and (**B**) 10 heme synthesis genes from *Synechococcus elongatus* PCC 7942. Symbols “**” indicate a highly significant difference compared with WT based on *p* < 0.01.

The results showed that most mutant strains showed significantly lower chlorophyll a contents than that of the WT (35.28 µg/mL), except for strain g-Ef (35.36 µg/mL) which showed insignificant variation in the content of chlorophyll a from that of the WT (Figure 6). The lowest chlorophyll a content was detected in strain g-Hn at 23.51 µg/mL.

### 2.6. Contents of Carotenoids in WT and Mutant Strains of Synechocystis sp. PCC 6803

**Figure 7:**
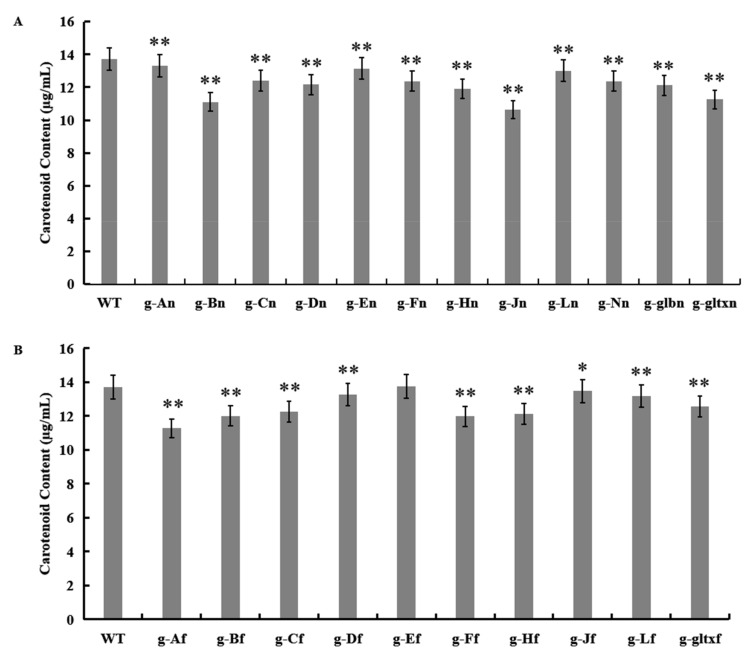
Contents of carotenoid in WT and mutant strains of *Synechocystis* sp. PCC 6803 based on the overexpression of (**A**) 12 heme synthesis genes from *Synechocystis* sp. PCC 6803 and (**B**) 10 heme synthesis genes from *Synechococcus elongatus* PCC 7942. The symbol “*” indicates a significant difference compared to WT (*p* < 0.05), and the symbol “**” indicates a highly significant difference compared to WT (*p* < 0.01).

The results showed that most mutant strains showed significantly lower carotenoid contents than that of the WT (13.71 µg/mL), except for strain g-Ef (13.75 µg/mL) which showed insignificant difference in the content of carotenoids from that of the WT (Figure 7). The lowest carotenoid content was detected in strain g-Jn (10.64 µg/mL).

### 2.7. Contents of Phycocyanin in WT and Mutant Strains of Synechocystis sp. PCC 6803

**Figure 8:**
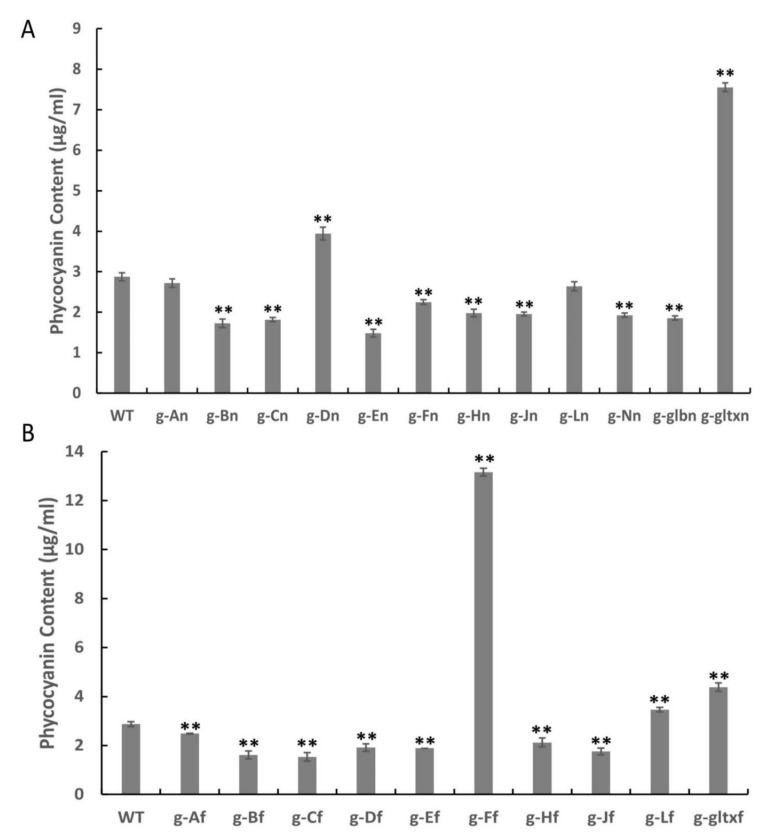
Contents of phycocyanin in WT and mutant strains of *Synechocystis* sp. PCC 6803 based on the overexpression of (**A**) 12 heme synthesis genes from *Synechocystis* sp. PCC 6803 and (**B**) 10 heme synthesis genes from *Synechococcus elongatus* PCC 7942. Symbols “**” indicate the significant difference compared with WT based on *p* < 0.01.

The results revealed significantly higher phycocyanin contents in a total of five mutant strains, including g-Dn (3.94 µg/mL), g-gltxn (7.55 µg/mL), g-Ff (13.16 µg/mL), g-Lf (3.46 mg/µL), and g-gltxf (4.38 µg/mL), in comparison with the WT (2.88 µg/mL), while the strain g-An (2.72 µg/mL) showed insignificant difference in the phycocyanin content from that of WT (Figure 8). Among them, g-Ff mutant strain had the highest phycocyanin content, reaching 4.57 times that of the WT. All other mutant strains showed significantly lower levels of phycocyanin than that of the WT, with the lowest phycocyanin content detected in strain g-En at 1.49 µg/mL.

### 2.8. Variations in Heme Gene Expression and Synthesis Pathway in Mutant Strains of Synechocystis sp. PCC 6803

**Figure 9:**
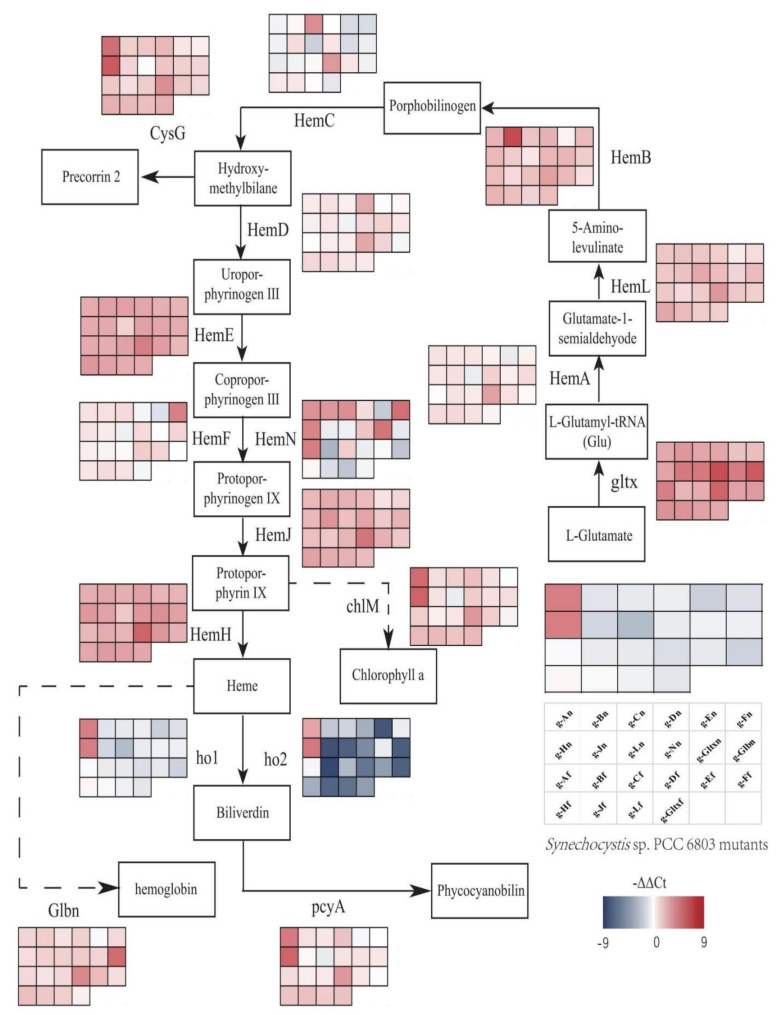
Gene expression profiles of heme synthesis pathways in mutant strains of *Synechocystis* sp. PCC 6803 based on the overexpression of 12 heme synthesis genes from *Synechocystis* sp. PCC 6803 and 10 heme synthesis genes from *Synechococcus elongatus* PCC 7942. The colour of the scale in the graph indicates the degree of up- and down-regulation of the gene. We used −ΔΔCt (−9 to 9) values corresponding to the colour of the scale (red indicates up-regulation, blue indicates down-regulation). The higher the value, the redder the colour, the higher the degree of gene upregulation. The lower the value, the darker the blue colour, the higher the degree of gene downregulation. Each cell of the table on the scale corresponds to a mutant strain (names are indicated in the table). The table next to each gene corresponds to the expression of this gene in the 22 mutant strains.

Variations in expression levels of heme synthesis genes and heme branching synthesis pathways were evaluated in all mutant strains based on qRT-PCR (Figure 9). According to Figure 9, the *gltx*, *hemB*, *hemL*, *hemE*, *hemH*, and *hemJ* genes were all up-regulated in all mutant strains compared to the wild strain. The *hemA*, *hemC*, *hemD*, *hemF*, and *hemN* genes were both up-regulated and down-regulated in different mutant strains. The *hemA* gene was only down-regulated in g-En, g-Ln mutant strains. The *hemC* gene was down-regulated in g-An, g-Dn, g-En, g-Fn, g-Hn, g-Ln, g-gltxn, g-glbn, g-Af, g-Bf, g-Ff, and g-gltxf. The *hemD* gene was only down-regulated in g-En, g-Ln, g-Af, and g-Ff mutant strains. The *hemF* gene was only down-regulated in g-Dn, g-En, g-Ln, g-Af, g-Ff, and g-gltxf mutant strains. The *hemN* gene was down-regulated in g-En, g-Jn, g-Ln, g-glbn, g-Bf, g-Df, g-Ef, g-Ff, g-Jf, g-Lf, and g-gltxf. Only the g-En mutant strain of the glbn gene was down-regulated. In the phycocyanin synthesis pathway, the ho1 gene was upregulated in g-An, g-Hn, and g-Hf mutant strains. Ho2 gene was upregulated in g-An, g-Hn mutant strains. The *pcyA* gene is down-regulated in g-En, g-Ln. In the other branching pathways, the *cysG* gene was down-regulated in g-Ln. *ChlM* was down-regulated in g-Fn and g-Ln. g-An and g-Hn resulted in significant up-regulation of all heme branching pathways, such as the chlorophyll synthesis gene *chlM*, the *ho1*, and *ho2* genes in the heme degradation pathway, and the phycocyanin synthesis gene *pcyA*, which was not observed in other mutant strains. This phenomenon was not observed in other mutant strains.

## 3. Discussion

The cell factories based on modified heterotrophic microorganisms as the chassis achieved high heme yields but suffered from many limitations such as high production costs and stringent requirements for both equipment and site, lacking the advantages of photosynthetic carbon sequestration and autotrophic release of oxygen, as shown in *Synechocystis* sp. PCC 6803. Furthermore, some microbes, such as *E. coli*, can themselves secrete endotoxins [24], severely limiting the use of the bioactive compounds synthesized, whereas varied pathways and regulatory mechanisms of heme synthesis are detected in different microbial taxa. To the best of our knowledge, the comprehensive studies of microalgae targeting the porphyrin synthesis pathway to facilitate the accumulation of both heme and phycocyanin are still not reported. Therefore, we used *Synechocystis* sp. PCC 6803 as a chassis cell to identify the potentially functional components of the heme synthesis pathway by overexpressing genes of both *Synechocystis* sp. PCC 6803 and *Synechococcus elongatus* PCC 7942 involved in heme synthesis pathway in strain PCC 6803 and to further explore the regulatory mechanisms underlying the productions of heme and phycocyanin. Overexpression of these genes may increase the metabolic flux of the heme and phycocyanin synthesis pathways, ultimately increasing the levels of heme and phycocyanin.

### 3.1. Enhanced Production of Heme in Mutant Strains of Synechocystis sp. PCC 6803

In recent years, investigations on the production of heme using microorganisms have been focused on heterotrophic microbes such as *E. coli*, *Corynebacterium glutamicum*, and yeast. For example, Kwon et al. reported that heme production was increased in *E. coli* by overexpression of various genes derived from *Rhodobacter capsulatum* (*hemA*), *E. coli* (*hemB*, *hemC*, *hemD*, and *hemF*), *Synechococcus* (*hemE*), and *Bacillus subtilis* (*hemY* and *hemH*) [25]. The heme production was further improved by the co-expression of HemA, NADP-dependent malic enzyme (MaeB), and dicarboxylate transporter protein (DctA) in *E. coli* [26]. Furthermore, Lee et al. found that the overexpression of pantothenate synthase resulted in a one-fold increase in heme production [27], while the overexpression of the gene involved in the porphyrin pathway (*hemBCDE*) showed a marginal effect on the productions of intermediates as well as heme and the expression of *hemC* enhanced the synthesis of ALA and uroporphyrin. Moreover, Zhao et al. applied metabolic engineering to modify the C5 pathway in *E. coli* and screen the overexpression targets in the heme biosynthesis pathway, adjusted the metabolic fluxes in heme biosynthesis, and disrupted the gene *yfeX* encoding a putative heme degrading enzyme, resulting in a heme yield of 7.88 mg/L [28]. Ko et al. applied systematic metabolic engineering and membrane surface modification in *Corynebacterium glutamicum* to block the degradation pathway of heme and preferentially direct the flow of precursors to the heme synthesis pathway, and to open the pathway for extracellular transfer, ultimately dramatically increasing the efficiency of heme synthesis in the cell factory [29]. Ishchuk et al. achieved a substantial increase in intracellular heme content in *Saccharomyces cerevisiae* by constructing a genome-scale metabolic model with combined modifications to the corresponding target genes based on computer simulations [30].

In our study, a total of 22 mutant strains based on *Synechocystis* sp. PCC 6803 were successfully constructed with overexpression of 12 heme synthesis genes of strain PCC 6803 and 10 heme synthesis genes of *Synechococcus elongatus* PCC 7942, respectively. Compared to WT, significantly higher heme content was detected in four mutant strains (i.e., g-glbn, g-Hn, g-Cn, and g-Fn), reaching 2.49, 1.38, 1.37, and 1.35 times the heme content of WT, respectively. The mutant strain with overexpression of *Synechocystis* sp. PCC 6803 endogenous haemoglobin (GlbN) showed the highest heme content. This was probably due to the ability of haemoglobin to bind heme and reduce the feedback inhibition and cytotoxicity of free heme on its synthetic pathway, ultimately increasing the total heme content in the microalgal cells. Similarly, Zhao et al. reported that by overexpressing the heme transporter protein CcmABC, excessive intracellular free heme content was prevented, thus increasing the total titer of cellular heme synthesis [28]. These results suggested that there was a strict regulatory mechanism regulating the intracellular free heme content. Furthermore, enhancing the metabolic sink of heme and maintaining the intracellular free heme content at the appropriate levels are the keys to constructing an engineered microbial strain for increased heme production. Pranawidjaja et al. showed that the conversion of PPIX to heme was the bottleneck step in heme biosynthesis [31]. These results were consistent with the findings revealed in our study. In particular, the overexpression of the enzyme (HemH) catalyzing the conversion of PPIX to heme in *Synechocystis* sp. PCC 6803 resulted in a significant increase in heme content and a significant decrease in chlorophyll content, suggesting that the overexpression of HemH promoted the PPIX flow to the heme synthesis pathway rather than its competing pathways, i.e., the chlorophyll synthesis pathway. The polymerization of four porphobilinogen molecules into 1-hydroxymethylbutane, catalyzed by HemC, is one of the key steps in the generation of heme [32,33]. Our results showed that the increased heme content was obtained in the mutant strain with overexpression of HemC in *Synechocystis* sp. PCC 6803. In addition, the overexpression of HemF also facilitated the accumulation of heme in *Synechocystis* sp. PCC 6803. These results were consistent with those previously reported, i.e., the overexpression of HemF was effective in enhancing the intracellular 5-ALA content [34], ultimately leading to the increased heme content in the mutant strain.

Although the heme contents of these mutant strains were high, their growth rates were mostly slower than that of the WT. This was probably due to either the cytotoxicity caused by excess free heme or the metabolic burden placed on the microalgal cells by the excessive accumulation of heme synthesis precursors. Our results revealed significantly lower heme contents in all other overexpression strains compared with the WT. It was speculated that this may be due to the overexpression of these enzymes leading to negative feedback inhibition effects of downstream products of the heme synthesis pathway, e.g., PPIX and heme, which in turn reduce the carbon flux of the heme synthesis pathway. It has been reported that the activities of HemA and HemB in *E. coli* are inhibited by feedback from downstream products [34], causing a decrease in the amount of 5-ALA, which is an important precursor for the synthesis of heme.

To explore the regulatory mechanisms of the heme and phycocyanin synthesis pathways in *Synechocystis* sp. PCC 6803, the expression patterns of genes involved in the heme and phycocyanin synthesis pathways were investigated in the WT and mutant strains of *Synechocystis* sp. PCC 6803. The results showed that *gltx*, *hemB*, *hemL*, *hemE*, *hemH*, and *hemJ* genes were all up-regulated in all mutants compared to WT. The *hemA* gene was up-regulated in all mutants except g-En, g-Ln. *hemC* gene was up-regulated in g-Bn, g-Cn, g-Jn, g-Nn, g-Cf, g-Df, g-Hf, g-Jf, and g-Lf. hemD gene was up-regulated in all mutants except g-En, g-Ln, g-Af, and g-Ff. *hemF* gene was up-regulated in all mutants except g-Dn, g-En, g-Ln, and g-Ff. The hemN gene was up-regulated in mutants except g-Dn, g-En, g-Ln, g-Af, g-Ff, and g-gltxf. *hemN* gene was up-regulated in mutants except g-An, g-Bn, g-Cn, g-Dn, g-Fn, g-Hn, g-Nn, g-gltxn, g-Af, g-Cf, and g-Hf. Glbn gene was up-regulated in mutants except g-En. The *glbN* gene was up-regulated in all mutants except the g-En mutant. In the heme branching pathway, the *ho1* gene was upregulated in g-An, g-Hn, and g-Hf mutant strains. *ho2* gene was upregulated in g-An, g-Hn mutant strains. *cysG* gene was downregulated in g-Ln. *chlM* gene was downregulated in g-Fn, g-Ln. *pcyA* gene was downregulated in g-En, g-Ln. g-An and g-Hn significantly upregulated the expression of all heme branching pathways, such as the chlorophyll synthesis gene *chlM*, the *ho1* and *ho2* genes in the heme degradation pathway, and the phycocyanin synthesis gene *pcyA*, which was not observed in other mutant strains, which seems to indicate that overexpression of hemH-syn and hemA-syn increases the flow of energy to porphyrin synthesis.

Previous studies showed that the overexpression of HemA caused the increased metabolic flux of the upstream pathway of heme synthesis [34], whereas our results showed that overexpression of *HemH-syn* promoted the accumulation of heme. Excess free heme in cells could cause damage to cells by the production of destructive ROS [35]. The heme oxygenase system is essential for the regulation of heme homeostasis [36]. Therefore, it was hypothesized that g-An, g-Hn, and g-Hf mutant strains regulated the intracellular heme content by up-regulating *ho1* or *ho2* to prevent cell damage caused by ROS. Studies showed that changes in the expression of individual genes in the heme synthesis pathway caused changes in the expression of genes in the entire synthesis pathway and the strength of the negative feedback effect of substrates, which ultimately affected the metabolic flux of the entire pathway [37]. Our results showed that the genes *cysG*, *chlM*, and *pcyA* involved in heme synthesis pathway were down-regulated in strain g-Ln with reduced heme content. HemL is the key enzyme catalyzing the synthesis of the key precursor of heme, 5-ALA. It was hypothesized that the overexpression of HemL caused feedback inhibition of heme and reduced carbon flux throughout the porphyrin synthesis pathway. In particular, the overexpression of *hemH-syf* resulted in the up-regulation of all the genes in the heme synthesis pathway of *Synechocystis* sp. PCC 6803, but no increase in the contents of heme and phycocyanin. The excessive amount of tetrapyrrole is usually toxic to cells. Therefore, organisms have strict control mechanisms over tetrapyrrole synthesis, including feedback inhibition and use of trans-acting factors in a variety of sophisticated regulatory approaches [38]. We speculate that the existence of a strict regulatory mechanism regulating the heme and phycocyanin syntheses in *Synechocystis* sp. PCC 6803 beyond the transcriptional level. Further studies are necessary to investigate the regulatory mechanisms of the enzymes involved in heme and phycocyanin synthesis pathways at the proteomic level.

### 3.2. Enhanced Production of Phycocyanin in Mutant Strains of Synechocystis sp. PCC 6803

Cyanobacteria, represented by *Spirulina*, are excellent hosts for the production of phycocyanin [39,40]. However, the *Spirulina* gene transformation system is not yet mature enough to make *Spirulina* an engineered host to further enhance the production of phycocyanin. It is well-known that *E. coli* is often used as an engineered host for heterologous production of phycocyanin [41,42,43]. However, the heterologous expression of phycocyanin has limitations of balanced synthesis and addition of chromophores as well as inefficient folding and complex assembly [44]. *Synechocystis* sp. PCC 6803 is rich in phycocyanin and contains a mature genetic transformation system [45], which is conducive to engineering modifications for high production of phycocyanin. Previous studies reported that the rate-limiting step for the synthesis of phycocyanin was catalyzed by Ho1 and PCYA [46,47,48]. However, these studies did not further explore the heme synthesis pathway, which is the upstream of phycocyanin synthesis. Therefore, we further investigated the effect of overexpression of genes related to the heme synthesis pathway on the production of phycocyanin in strain PCC 6803. Our results showed that the significantly higher levels of phycocyanin were detected in mutant strains g-gltxn, g-gltxf, g-Dn, g-Ff, and g-Lf, reaching 2.62, 1.52, 1.37, 4.58, 1.2 times of that of WT, respectively. As the first enzyme in the heme synthesis pathway, GltX catalyzes the linkage of glutamate with tRNA^Glu^, suggesting that the overexpression of *gltx* increased the energy flow throughout the pathway, leading to an increase in the content of phycocyanin. Studies showed that the overexpression of *hemD*, *hemF*, and *hemL* caused the increased 5-ALA content in cells [34,49,50], which was probably an important factor to increase the content of phycocyanin. Furthermore, our results revealed varied effects of different sources of heme synthesis pathway-related isozymes on heme and phycocyanin synthesis in *Synechocystis* sp. PCC 6803. The metabolically engineered *E. coli* was able to reach a heme content of 301.9 μg/g DW [29]. This was higher than the maximum heme content of 56.65 μg/g DW in the PCC 6803 mutant strain (g-glbn) that we obtained in our study. The wild-type *Spirulina* phycocyanin content was reported to reach 228 µg/mL [51], which was higher than the maximum yield of 13.16 µg/mL of phycocyanin obtained from our PCC 6803 mutant strain (g-Ff). Therefore, although our study provided crucial genetic targets for increasing the heme and phycocyanin contents in *Synechocystis* sp. PCC 6803, further metabolic engineering is needed to obtain the desired heme and phycocyanin production.

## 4. Materials and Methods

### 4.1. Microalgal Strains and Growth Conditions

*Synechocystis* sp. PCC 6803 and *Synechococcus elongatus* PCC 7942 were purchased from the Freshwater Algae Culture Collection at the Institute of Hydrobiology (FACHB), Chinese Academy of Sciences (Wuhan, China), designated as FACHB-898 and FACHB-805, respectively. Both strains were able to grow in both seawater and freshwater environment [22,52]. The mutant strains and WT of strain PCC 6803 were selected and cultured in liquid BG-l1 medium to the logarithmic phase with the OD_730_ of the initial inoculation adjusted to 0.05. Samples were taken every 48 h with the OD_730_ values measured by SpectraMax M2 (Molecular Devices, CA, USA) to evaluate the growth of the strains. The growth curves were generated based on three biological replicates performed in each of the experimental and the control groups. The mutant strains and WT of strain PCC 6803 were cultured in liquid BG-l1 medium to the late logarithmic phase and the cells were collected by centrifugation at 4000 rpm for 10 min to measure the contents of various substances in these strains of PCC 6803.

### 4.2. Vector Construction

The structure of the plasmid is shown in Supplementary Figure S1. PCR analysis based on the genome of strain PCC 6803 was performed using primers Pcpc560-F and Pcpc560-R to obtain the constitutive promoter *Pcpc560*, which was a 560-bp fragment upstream of the translation start site of the *cpcB* gene in strain PCC 6803 [53]. Primers Pnrsb-F and Pnrsb-R were used to amplify the genome of strain PCC 6803 to obtain the inducible promoter P*nrsB* [54]. Both primers T1T2-F and T1T2-R were used to amplify the genome of *Escherichia coli* DH5-α to obtain the T1T2 terminator (GenBank accession No. LC703879.1). Primer pairs NSC2U-F/NSC2U-R and NSC2D-F/NSC2D-R were used to amplify the genome of strain PCC 6803 to obtain the upstream and downstream arms of neutral site 2 (NSC2) of strain PCC 6803; primer pairs NSC1U-F/NSC1U-R and NSC1D-F/NSC1D-R were used to obtain the upstream and downstream arms of NSC1 of strain PCC 6803 [55]. Primer pairs Cm-F/Cm-R and Gm-F/Gm-R were used to amplify the laboratory-preserved pBlunt-Cm and pBlunt-Gm vectors to obtain the chloramphenicol resistance gene expression cassette (Cm) and the gentamicin resistance gene expression cassette (Gm), respectively. The gene sequences of strains PCC 6803 and PCC 7942 were queried using the National Center for Biotechnology Information (NCBI) database (https://www.ncbi.nlm.nih.gov/, accessed on 16 March 2022). The genes in the heme synthesis pathway of strains PCC 6803 and PCC 7942 and the haemoglobin gene (*glbn*) of strain PCC 6803 were obtained by PCR using primers given in Table S3.

Using plasmid pBlueScript II KS (+) (PBSK; Fenghui Biology, Hunan, China) as the starting vector, the fragments of PBSK and T1T2 were digested and linked by *EcoR* I and *Pst* I to obtain plasmid PBSK+T1T2. The fragments of PBSK+T1T2, NSC1D, and NSC2D were digested and linked with *Sac* I and *Not* I to obtain plasmids PBSK+T1T2+1D and PBSK+T1T2+2D, respectively. The fragments of PBSK+T1T2+1D, PBSK+T1T2+2D, NSC1U, and NSC2U were digested and linked with *Kpn* I and *Xho* I to obtain plasmids PBSK+T1T2+2UD and PBSK+T1T2+1UD, respectively. The fragments of PBSK+T1T2+2UD, PBSK+T1T2 +1UD, Cm (GenBank accession No. ADC79570.1), and Gm (GenBank accession No. CAA10280.1) were digested and ligated with *BamH* I to obtain plasmids 2UD+Cm and 1UD+Gm, respectively.

The 10 endogenous heme genes of strain PCC 6803, including hemA-syn (slr1808), hemB-syn (sll1994), hemC-syn (slr1887), hemE-syn (slr0536), hemF-syn (sll1185), hemH-syn (slr0839), hemJ-syn (slr1790), hemL-syn (sll0017), hemN-syn (sll1876), and glbn (slr2097), were amplified by PCR, and the promoter Pcpc560 (P) was connected with each of the heme genes to obtain the overlapped fragment P+gene. The 10 overlapped fragments (i.e., P+hemA-syn, P+hemB-syn, P+hemC-syn, P+hemE-syn, P+hemF-syn, P+hemH-syn, P+hemJ-syn, P+hemL-syn, P+hemN-syn, and P+glbn) were digested by EcoR I and Sal I, and the overexpression plasmids of cytoplasmic framework 2UD+Cm and 1UD+Gm were digested by EcoR I and Sal I. The overexpression plasmids of heme synthesis genes of strain PCC 6803 were obtained by ligation and transformation (Table S4). The PCR product was obtained by inverse PCR overexpression of the plasmid framework 2UD+Cm using primers 2udPT-F and 2udPT-R. The 8 heme genes of strain PCC 7942, including hemA-syf (Synpcc7942_0504), hemB-syf (Synpcc7942_1792), hemC-syf (Synpcc7942_0967), hemE-syf (Synpcc7942_1086), hemF-syf (Synpcc7942_0674), hemH-syf (Synpcc7942_0137), hemJ-syf (Synpcc7942_0849), and hemL-syf (Synpcc7942_0645), were homologously recombined to obtain the overexpression plasmids of strain PCC 7942 heme synthesis genes. The two endogenous genes of strain PCC 6803, i.e., hemD-syn (sll0166) and gltx-syn (sll0179), and the two genes of strain PCC 7942, i.e., hemD-syf (Synpcc7942_0272) and gltx-syf (Synpcc7942_2393), were driven by the inducible promoter PnrsB. Primer pair IP-F/IP-gn-R was used to amplify the promoter driving gltx-syn, primer pair IP-F/IP-dn-R used to amplify the promoter driving hemD-syn, primer pair IP-F/IP-gf-R used to amplify the promoter driving gltx-syf, and primer pair IP-F/IP-df-R used to amplify the promoter driving hemD-syn. Primers 2u-noP-F and 2udPT-F were used to amplify the overexpression plasmid framework 2UD+Cm to obtain a promoterless framework, and four PnrsB overexpression plasmids were obtained based on the homologous recombination of these four genes, including hemD-syn, gltx-syn, hemD-syf, and gltx-syf.

### 4.3. Construction of the Overexpression Mutants of Synechocystis sp. PCC6803

The natural transformation of strain PCC 6803 was performed based on the previous study [56], with minor modifications. Cells in the logarithmic growth phase (OD_730_ = 0.6−0.8) were collected by centrifugation at 4000 rpm for 10 min and washed three times with fresh BG-11 liquid medium. A total of 20 μL homologous recombination vector was added to 1 mL of sample solution and incubated for 6 h under low light. Then, the mixture was evenly spread in a BG-11 plate with a mixed cellulose ester membrane (Jinteng, Tianjin, China, JTMF00502). In 24 h, the mixed cellulose ester membrane was placed on a BG-11 plate containing 15 μg/mL chloramphenicol or 15 μg/mL gentamicin. The transformed strain PCC 6803 was incubated for two weeks at 30 °C in an incubator with a light intensity of 2000 lux and a light/dark photoperiod of 12-h/12-h. A single colony was picked on the BG-11 plate containing chloramphenicol for continuous sub-screening and to finally expand the transformants in BG-11 liquid medium with 50 μg/mL chloramphenicol or 20 μg/mL gentamicin. The sample fluid was collected by centrifugation at 4000 rpm for 10 min. Genomic DNA was extracted from strains PCC 6803 using the lysate method described by Singh et al. [57]. The *g-glbn* mutant and WT of strain PCC 6803 were amplified using primers NSC1U-F and NSC1D-R, and other transformants and WT of strain PCC 6803 were amplified using primers NSC2U-F and NSC2D-R. Successful transformants of strain PCC 6803 were identified by comparison with the size of the WT bands of electrophoresis.

### 4.4. Protein Isolation and Western Blot Analysis

The WT and mutant cyanobacterial cells in the logarithmic growth phase were collected by centrifugation at 4000 rpm for 10 min and resuspended in Tris-HCl (40 mM and pH 8.0), with the protease inhibitor PMSF (Yuanye, Shanghai, China) added at the final concentration of 1.5 mM. The microalgal cells were sonicated in an ice bath and centrifuged, and the supernatant was aspirated to obtain the total protein of the microalgal cells. Protein concentrations were determined using BCA Protein Assay Kit (Vazyme, Nanjing, China). Western blot analysis was performed using the standard methods. We used the SDS-PAGE Gel Rapid Kit (Beyotime, Shanghai, China, P0012AC) to prepare the protein gels. Briefly, a total of 7 mL of the lower gel was placed in a beaker with 70 μL of procoagulant added, mixed well, and added to the gel-making glass plate, then added with 1 mL of sterile water and sealed for 20 min. The sealing solution was tilted and poured, and the sterile water was absorbed using the filter paper. Two mL of the top layer of glue were moved into a beaker and added with 20 μL of coagulant, mixed well, and added to the gel-making glass plate; a comb was inserted and waited for 20 min. Protein samples (each of 50 μg) and an equal volume of 2× SDS loading buffer were added, boiled for 10 min, and then centrifuged at 12,000 rpm for 10 min. The supernatant was the desired protein sample. A total of 50 μg of protein sample and 5 μL of marker were added to each lane of electrophoresis and run at 70 V for 30 min with constant pressure, then 120 V for 1.5 h at constant pressure, and waited for the bromophenol blue band to run out of the lower gel layer to stop the electrophoresis. They were then transferred to the polyvinylidene difluoride (PVDF) membranes (Solarbio, Beijing, China) at 70 V, and blocked with 5% nonfat milk powder at room temperature for 1 h. Then, the PVDF membranes were removed and washed 3 times with Tris-buffered saline (TBS) containing 0.1% (*v*/*v*) Tween-20 (TBST) for 5 min with shaking, 5 times in total. A total of 10 μL of His Tag Mouse Monoclonal Antibody (Beyotime, Shanghai, China; AF5060) was added to 10 mL of the blocking solution, mixed well, with the PVDF membrane incubated on a shaking table at 20 ℃ for 3 h. Then, the PVDF membrane was placed in TBST for 5 min with shaking, washed for a total of 5 times. A total of 10 μL of HRP-labelled Goat Anti-Mouse IgG (H+L) (Beyotime, Shanghai, China; A0216) was added to 10 mL of the blocking solution, mixed well, and the PVDF membrane was incubated on a shaking table for 1 h. Then, the PVDF membrane was placed in TBST for 5 min with shaking to wash for 5 times and was then stained with ECL Chemiluminescent Substrate Reagent Kit (Vazyme, Nanjing, China, ID, E412-01).

### 4.5. RNA Extraction and cDNA Synthesis

Both WT and transformant cell lines were cultured and harvested during the exponential growth phase, with the total RNA isolated from the samples using RNA-easy Isolation Reagent (Vazyme, Nanjing, China) following the manufacturer’s instructions. First-strand cDNA was synthesized using HiScript Q RT SuperMix for qPCR (+gDNA wiper) (Vazyme, Nanjing, China) following the manufacturer’s instructions (Reverse transcription using 1 µg of RNA per sample tube).

### 4.6. Quantitative Reverse-Transcription PCR Analysis

Quantitative reverse-transcription PCR (qRT-PCR) analysis of heme synthesis gene expression was performed using the CFX Connect Real-Time PCR System (Bio-Rad, Hercules, CA, USA). Primers rnpB-F and rnpB-R were used to amplify *rnpB*, which was used as an internal reference gene. Primers (Table S5) were designed to amplify the heme synthesis genes with the reactions prepared following the manufacturer’s instructions. Each reaction was repeated with three biological replicates in a total volume of 20 μL containing 10 μL of ChamQ Universal SYBR qPCR Master Mix (Vazyme, Nanjing, China), 200 nM of each primer, and 1 μL of 10-fold diluted cDNA template. Reactions were performed in 8-well optical grade PCR plates, with the following amplification procedures: initial denaturation at 95 °C for 30 s, followed by 40 cycles of 95 °C for 10 s and 60 °C for 30 s, with the melting curves generated using Bio-Rad CFX Maestro 2.2 Version 5.2.008.0222 (Bio-Rad, CA, USA). Sterile water, instead of cDNA template, was used in the negative control. The relative expression levels of heme synthesis genes were determined using the relative 2^−ΔΔCt^ method [58].

### 4.7. Contents of Heme in Synechocystis sp. PCC6803

First, cells of strain PCC 6803 (three biological replicates per group) were harvested by centrifugation at 12,000× *g* for 5 min, with the supernatant removed. The algal cells were washed three time each with sterile water and the precipitate was collected by centrifugation at 12,000× *g*/min for 5 min. The sample lyophilized in a freeze dryer. The freeze-dried powder (0.01 g) of strain PCC 6803 was accurately weighed and added with 80% neutral acetone to extract chlorophyll a [59]. The mixture was centrifuged, the supernatant removed, and repeatedly extracted using 80% neutral acetone until the supernatant was no longer green. The fluorometric method was used to detect the heme content in the microalgae strain [60]. The specific steps are 20 mM oxalic acid was added to the obtained precipitate and placed at 4 ℃ for 24 h, and then sonicated. After adding 2 M oxalic acid, it was divided into two equal parts, one was heated at 98 ℃ for 30 min, and the other was placed at room temperature for 30 min. It was added to the black 96 well plate, and the fluorescence value was measured under the excitation light of 400 nm and the emission light of 620 nm. The fluorescence value placed at room temperature represents the content of endogenous porphyrins in *Synechocystis* sp. PCC 6803, and the fluorescence value heated at 98 ℃ represents the content of heme and endogenous porphyrins. The heme solution prepared with 1% (*w*/*v*) bovine serum albumin and 0.01 N KOH was added to the oxalic acid solution, heated to prepare the standard, and the standard curve was drawn.

### 4.8. Contents of Chlorophyll A and Carotenoids in Synechocystis sp. PCC 6803

The contents of chlorophyll a and carotenoids were measured according to the studies previously reported [61]. The appropriate microalgal liquid was collected and centrifuged at 12,000× *g* for 5 min, with the supernatant removed. The sample was added with 80% neutral acetone to extract the pigments at 55 °C for 30 min and centrifuged at 12,000 g for 5 min. The colour of the precipitation was observed, i.e., if the precipitation was still green, then the centrifugation was repeated until the precipitation was blue. The 80% neutral acetone was used as blank to calibrate the spectrophotometer (SHIMADZU, Kyoto, Japan), which was used to measure the absorbance values of the sample and blank at 470 nm, 665 nm, and 720 nm, respectively. The content of chlorophyll a was calculated according to Equation (1) and the content of carotenoids was calculated according to Equations (1) and (2)

Chla (µg/mL) = 12.9447 × (A665 − A720)(1)

carotenoids (µg/mL) = [1000 × (A470 − A720) − 2.86 × Chla (µg/mL)]/221(2)

### 4.9. Content of Phycocyanin in Synechocystis sp. PCC 6803

The content of phycocyanin was determined using the methods previously reported [62]. A total of 20 mg freeze-dried microalgal powder was resuspend with 1 mL phosphate buffer (0.05 mol/L and pH = 6.8). The mixture was frozen at −80 °C for 24 h and was then slowly thawed in a constant temperature metal bath at 4 °C. The dissolved sample solution was ultrasonically broken. The crude extract was centrifuged at 12,000× *g* for 10 min to collect the supernatant, which was used to measure the absorbance values at 615 nm and 652 nm, respectively, using the spectrophotometer. The content of phycocyanin (C-PC) was determined according to Formula (3).

phycocyanin (mg/mL) = (A615 − 0.474 × A652)/5.34(3)

### 4.10. Statistical Analysis

Data were presented as means ± standard deviation (SD) based on three biological replicates. Data were statistically analyzed by SPSS 17.0 (SPSS Inc., Chicago, IL, USA). The one-way ANOVA (LSD) was performed to determine the significant differences among treatments based on *p* < 0.05 or *p* < 0.01.

## 5. Conclusions

In the present study, overexpression of genes in the porphyrin pathway derived from *Synechocystis* sp. PCC 6803 or *Synechococcus elongatus* PCC 7942 promoted the accumulation of heme and phycocyanin in strain PCC 6803. To explore the underlying mechanism, we investigated a series of physiological and biological properties of wild type and mutant strains of PCC 6803. By studying the transcriptional expression levels of related genes, we further explored the molecular mechanism of enhancing heme and phycocyanin and provided a new strategy to promote heme and phycocyanin accumulation. Thus, genetic engineering strategies based on metabolic modifications of porphyrin pathway-related genes provide a broad prospect for establishing *Synechocystis* sp. PCC 6803 as a cellular factory for heme and phycocyanin productions.

## Data Availability

All raw data are readily available upon request.

## Supplementary Materials

The following supporting information can be downloaded at: https://www.mdpi.com/article/10.3390/md21070403/s1, Figure S1. Gene expression vector constructions. Table S1. The measured amount in Figure 5, Figure 6, Figure 7 and Figure 8; the length of the expected DNA fragments in Figure 2. Table S2. The reader OD_730_ of Figure 4. Table S3: Primers for PCR in this study. Table S4: Gene overexpression vector ligation and transformation system in *Synechocystis* sp. PCC 6803. Table S5: Primers and their sequences used for qRT-PCR in this study. Table S6: Molecular weights of target proteins overexpressed in the mutant strains of *Synechocystis* sp. PCC 6803 derived from either strain PCC 6803 or *Synechococcus* elongatus PCC 7942.
