# Supplementary material for: Promoting Heme and Phycocyanin Biosynthesis in Synechocystis sp. PCC 6803 by Overexpression of Porphyrin Pathway Genes with Genetic Engineering
Source: Mar Drugs. 2023 Jul 13;21(7):403. doi: 10.3390/md21070403 (PMC10382063; doi:10.3390/md21070403)
Supplement: Supplementary file 1 [file marinedrugs-21-00403-s001.zip › marinedrugs-2468134-supplementary.pdf]

marine drugs

MDPI

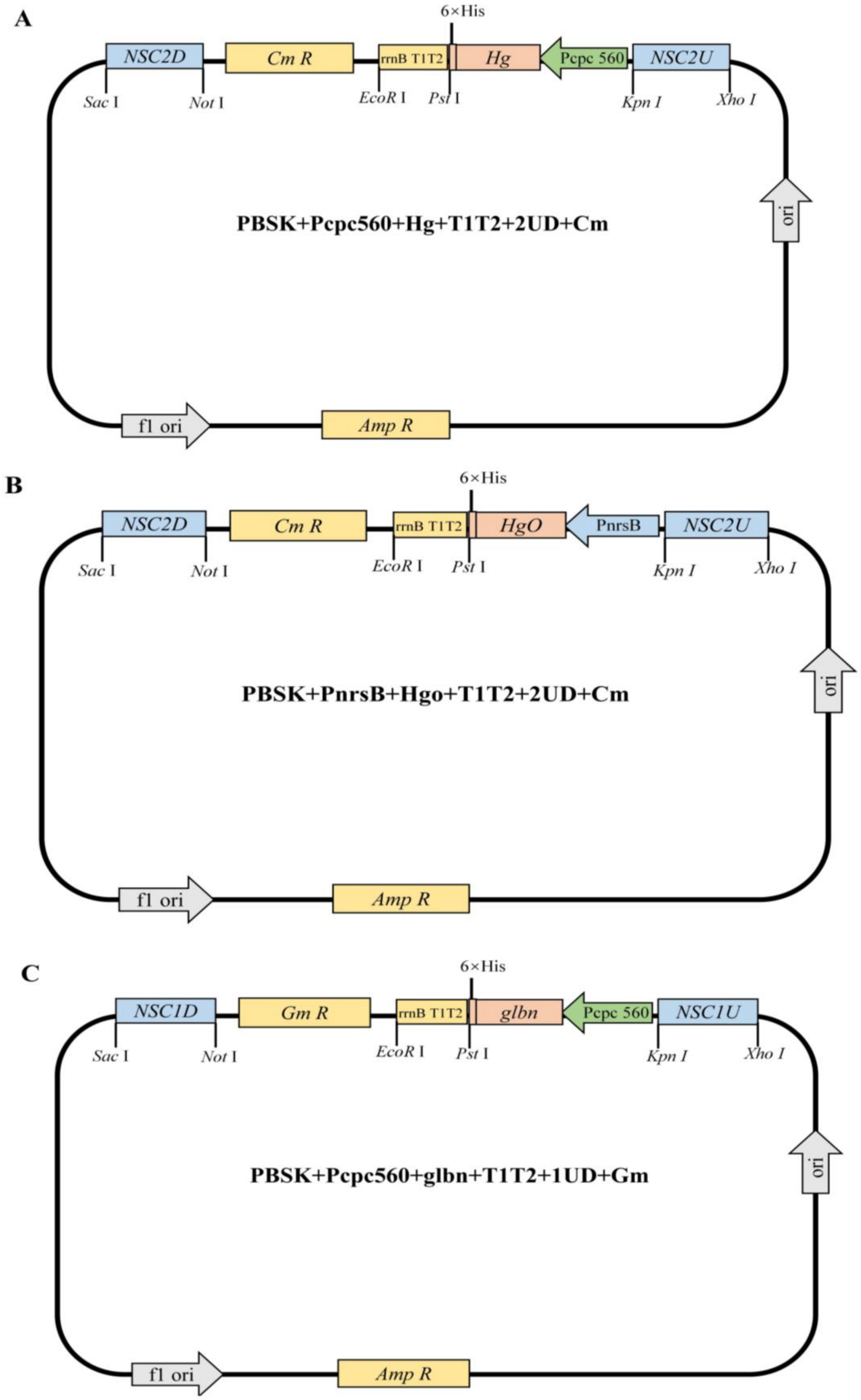

Figure S1. Gene expression vector constructions. The nsc1u/nsc1d and nsc2u/nsc2d are the upstream/downstream homologous arms of the expression vectors. CmR, Gm, and AmpR are the chloramphenicol, gentamicin, an ampicillin resistance expression cassettes, respectively. RrnB T1T2 is

the terminator, Pcpc560 is the promoter, and pnrsB is the inducible promoter. The expression vector of Figure A is used to insert PCC 6803 endogenous genes (i.e., *hemA*, *hemB*, *hemC*, *hemE*, *hemF*, *hemH*,*hemJ*, *hemL*, and *hemN*) and PCC 7942 genes (i.e., *hemA*, *hemB*, *hemC*, *hemE*, *hemF*, *hemH*, *hemJ*, and *hemL*) at the position of Hg, respectively. Genes of PCC 6803 and PCC 7942 origins (hemD and gltx) are inserted at the position of Hgo, respectively, using the expression vector in Figure B. Gene *glbn* encodes hemoglobin in PCC 6803.

Table S1. The measured amount in the Figures 5–8; the length of the expected DNA fragments in Figure 2.

	**Figure 5:** Heme content (ug/g)	**Figure 6:** Chlorophyll a content (ug/ml)	**Figure 7:** Carotenoid Content (ug/ml)	**Figure 8:** Phycocyanin content (ug/ml)	**Figure 2:** length of DNA fragments (bp)
WT	22.64	35.28	13.71	2.89	2329
g-An	4.51	34.70	13.31	2.72	5159
g-Bn	3.48	29.63	11.10	1.72	4859
g-Cn	31.51	32.62	12.41	1.82	4838
g-Dn	6.30	32.29	12.17	3.94	4992
g-En	6.99	34.09	13.14	1.49	4928
g-Fn	31.09	32.45	12.37	2.25	4898
g-Hn	31.53	23.51	11.92	1.98	4981
g-Jn	8.73	28.77	10.64	1.96	4399
g-Ln	5.50	34.12	13.02	2.64	5119
g-Nn	5.52	32.72	12.38	1.93	5218
g-gltxn	5.92	30.18	11.26	7.55	4627
g-glbn	56.65	32.25	12.12	1.86	4472
WT	22.64	35.28	13.71	2.89	2329
g-Af	7.96	30.48	11.28	2.49	5128
g-Bf	10.23	31.95	12.01	1.61	4792
g-Cf	7.55	32.46	12.26	1.53	4774
g-Df	3.05	34.50	13.26	1.92	4180
g-Ef	5.52	35.36	13.75	1.89	4876
g-Ff	5.03	31.99	11.99	13.16	4765
g-Hf	5.00	32.28	12.12	2.12	4975
g-Jf	3.93	34.83	13.48	1.75	4423
g-Lf	3.74	34.28	13.17	3.46	5173
g-gltxf	6.90	33.11	12.58	4.38	4821

Table S2. The readed OD_{730} of the Figure 4.

Days/Strains	0	2	4	6	8	10	12	14
g-An	0.005	0.021	0.076	0.154	0.203	0.302	0.374	0.445
g-Bn	0.005	0.032	0.087	0.156	0.252	0.406	0.451	0.542
g-Cn	0.005	0.020	0.087	0.158	0.232	0.318	0.405	0.444
g-Dn	0.005	0.012	0.012	0.011	0.011	0.007	0.008	0.009
g-En	0.005	0.014	0.042	0.104	0.154	0.224	0.344	0.392
g-Fn	0.005	0.027	0.048	0.071	0.085	0.113	0.135	0.152
g-Hn	0.005	0.029	0.067	0.115	0.183	0.234	0.325	0.382
g-Ln	0.005	0.019	0.071	0.118	0.227	0.282	0.361	0.471
g-Jn	0.005	0.024	0.090	0.144	0.212	0.341	0.406	0.497
g-Nn	0.005	0.026	0.058	0.123	0.204	0.301	0.396	0.487
g-glbn	0.005	0.022	0.039	0.079	0.133	0.129	0.224	0.249
g-gltxn	0.005	0.013	0.014	0.011	0.011	0.010	0.009	0.010
WT	0.005	0.028	0.093	0.178	0.292	0.385	0.474	0.528
g-Af	0.005	0.018	0.079	0.126	0.217	0.325	0.392	0.492
g-Bf	0.005	0.023	0.096	0.174	0.304	0.517	0.525	0.631
g-Cf	0.005	0.029	0.073	0.176	0.203	0.318	0.409	0.499
g-Df	0.005	0.008	0.009	0.012	0.006	0.007	0.006	0.005
g-Ef	0.005	0.017	0.063	0.127	0.214	0.428	0.426	0.493
g-Ff	0.005	0.047	0.076	0.144	0.190	0.331	0.383	0.540
g-Hf	0.005	0.019	0.081	0.132	0.212	0.316	0.373	0.425
g-Jf	0.005	0.028	0.071	0.149	0.246	0.371	0.428	0.471
g-Lf	0.005	0.024	0.074	0.138	0.204	0.306	0.419	0.597
g-gltxf	0.005	0.008	0.006	0.006	0.006	0.006	0.006	0.005

Table S3. Primers and their sequences used for PCR in this study. Letters "F" and "R" present the forward and reverse primers, respectively. Sequences of the homologous arms used in homologous recombination are indicated in lowercase letters.

Primer	Primer sequence (5′→3′)
Pcpc560-F	gatgtcgacACCTGTAGAGAAGAGTCCCT
Pcpc560-R	TGAATTAATCTCCTACTTGAC
T1T2-F	gatgaattcCTGTTTTGGCGGATGAGAGAA
T1T2-R	gatctgcagAAGAGTTTGTAGAAACGCAAAAAGG
Pnrsb-F	TTCCACCAGCAAAATTCGCATC
Pnrsb-R	ACCACCTCAAATTGGGAAT
NSC1U-F	gatggtaccTTAACAGCAGAAAAAACCGTTGC
NSC1U-R	gatctcgagTTCTCCCATGTTGAAAAAGTCCC
NSC1D-F	gatgcggccgcTTTAACAATTTGCCATGGGCA
NSC1D-R	gatgagctcTTGTCTCCTTGGTTGAAATTTATGG
NSC2U-F	gatggtaccGCGATATATTGGTGAAGCAATTCC

NSC2U-R	gatctcgagCTGTATGGAATCAAATCGATAGTTTCC
NSC2D-F	gatgcggccgcAATTCCTTGACTAGGGCTAAAAATAGG
NSC2D-R	gatgagctcTAAATTACCCTGGCAGGAATGG
Cm-F	gatggatccTGATCGGCACGTAAGAGGTTC
Cm-R	gatggatccAATAGACATAAGCGGCTATTTAACGA
Gm-F	gatggatccTCACCATTTGGACAAAACATCAG
Gm-R	gatggatccAAACGCAAAAGAAAATGCCG
Hema-syn-F	AGTCAAGTAGGAGATTAATTCAGTGGTCTGCCATTCGGACTACA
Hema-syn-R	gatgaattcCTAATGATGATGATGATGATGACCGAATTGTTCTTCCACTTCC
Hemb-syn-F	AGTCAAGTAGGAGATTAATTCAATGTTTCCCACCATCCGTCC
Hemb-syn-R	gatgaattcCTAATGATGATGATGATGATGGTCCTGTAGCCAGCGGGC
Hemc-syn-F	AGTCAAGTAGGAGATTAATTCAATGACTGTTTCGACCTCTGCTCC
Hemc-syn-R	gatgaattcTTAATGATGATGATGATGATGTCCCCGGCCAGCTTC
Hemd-syn-F	CCCAATTTGAGGTGGTATGGCTGAAAAACTACCTCACCCAC
Hemd-syn-R	TAATGATGATGATGATGATGCGGATTTTGGTAAACATAATTAGTAATG
Heme-syn-F	AGTCAAGTAGGAGATTAATTCAATGACAGAAGCAAATGATTTGCC
Heme-syn-R	gatgaattcTTAATGATGATGATGATGATGCAGTAATTGATCCACTTGCTTAGC
Hemf-syn-F	AGTCAAGTAGGAGATTAATTCAATGACCGTCTCTCCCACAACC
Hemf-syn-R	gatgaattcCTAATGATGATGATGATGATGACTATTAACCCAGTCCTGGGG
Hemh-syn-F	AGTCAAGTAGGAGATTAATTCAATGGGTCGTGTTGGGGTCTT
Hemh-syn-R	gatgaattcCTAATGATGATGATGATGATGAAGCAAGCCGACAAAATGC
Hemj-syn-F	AGTCAAGTAGGAGATTAATTCAATGGCCTACTACTGGTTTAAAGCC
Hemj-syn-R	gatgaattcCTAATGATGATGATGATGATGATTCTGAGCAGAAGCCGCT
Heml-syn-F	AGTCAAGTAGGAGATTAATTCATTGGTTAACGCAACCCCTTTT
Heml-syn-R	gatgaattcTCAATGATGATGATGATGATGGAGAGTAGCAAATACTTCCTTAGC
Hemn-syn-F	AGTCAAGTAGGAGATTAATTCAATGACCACCCACATTTCCTACTGTTG
Hemn-syn-R	gatgaattcTTAATGATGATGATGATGATGAATTGCCCGAGAAAACATCT
gltx-syn-F	TTCCCAATTTGAGGTGGTGTGACTGTCCGTGTCCGCATTGC
gltx-syn-R	TAATGATGATGATGATGATGTTCAGCGGCGATCGCCTG
Glbn-syn-F	AGTCAAGTAGGAGATTAATTCAATGTCAACTTTGTATGAAAAATTAGGTG
Glbn-syn-R	gatgaattcTCAATGATGATGATGATGATGCTGATTAAGCACGTCCCG
Hema-syf-F	tcaagtaggagattaattcaATGCATCTCGCAGTTGTCGG
Hema-syf-R	taatgatgatgatgatgatgGCTACTCAACTGTCCTTGTGGATCG
Hemb-syf-F	tcaagtaggagattaattcaATGTTCCCCACGCATCGTC
Hemb-syf-R	taatgatgatgatgatgatgGGCGAGCCAGCGAGCTGC
Hemc-syf-F	tcaagtaggagattaattcaATGGTCTCCAGCCCTGCC
Hemc-syf-R	taatgatgatgatgatgatgAGCTTCAGGGCGAACAGTCG
Hemd-syf-F	TCCCAATTTGAGGTGGTATGGCTGAGCAGCCGCTGATCGGT
Hemd-syf-R	TAATGATGATGATGATGATGCGTGGTTTGGCGCGCCCA
Heme-syf-F	tcaagtaggagattaattcaATGGTCGCGTCGTCTTCGC

Heme-syf-R	taatgatgatgatgatgatgGTGACTCGCTGCCAAGAGTTG
Hemf-syf-F	tcaagtaggagattaattcaATGCAGACCCTGCAAGACGA
Hemf-syf-R	taatgatgatgatgatgatgGGCGGTCGGCCAGTTGAC
Hemh-syf-F	tcaagtaggagattaattcaATGGGGCGCGTCGGCGTC
Hemh-syf-R	taatgatgatgatgatgatgGAGCAACCCCAAGGCATGC
Hemj-syf-F	tcaagtaggagattaattcaTTGCGCTCTCTTGAGGCCG
Hemj-syf-R	taatgatgatgatgatgatgGGACTGGGTGGGCGCTGC
Heml-syf-F	tcaagtaggagattaattcaATGAGGAGGCTGTCAGCATCG
Heml-syf-R	taatgatgatgatgatgatgCAGCGCACTCATCACCGTACG
gltx-syf-F	TCCCAATTTGAGGTGGTGTGTCAGTTCGCGTTCGCATTGCTC
gltx-syf-R	TAATGATGATGATGATGATGGCCCTGAGCCGCCGCGAT
2udPT-F	CATCATCATCATCATCATTAGgaattcCTGTTTTGGCGGATGAGAGAAGA
2udPT-R	TGAATTAATCTCCTACTTGACTTTATGAGT
2u-noP-F	CTGTATGGAATCAAATCGATAGTTTC
IP-F	ATCGATTTGATTCCATACAGTTCCACCAGCAAAATTCGCA
IP-gn-R	CGGACAGTCACACCACCTCAAATTGGGAATTTGTCCAAGAT
IP-dn-R	GTTTTTCAGCCATACCACCTCAAATTGGGAATTTGTCCAAGAT
IP-gf-R	GCGAACTGACACACCACCTCAAATTGGGAATTTGTCCAAGAT
IP-df-R	CTGCTCAGCCATACCACCTCAAATTGGGAATTTGTCCAAGAT

Table S4. Gene overexpression vector ligation and transformation system in *Synechocystis* sp. PCC 6803. The system was configured and ligated with T4 ligase at 16 °C for 12 h (Supplementary Table 4). The ligation product was transformed into *E. coli* as follows. After a brief incubation in ice, the mixture of *E. coli* receptor cells and DNA was placed at 42 °C for 45 sec (heat excitation) and then placed back in ice. The LB medium was added and the transformed cells were incubated at 37 °C for 30 min with agitation. The bacterial solution was applied to LB solid plates with relevant resistance using a pre-sterilized applicator. Single clones on the screening plate were picked for colony PCR to screen positive transformants. The positive transformants were sequenced (Tsingke, Beijing, China) and then expanded to culture with the plasmids extracted using the Plasmid Extraction Kit (Vazyme, Nanjing, China; DC201-01).

Name	Volume
Carrier enzyme section	1 μL
Gene fragments	7 μL
T4 DNA ligase	1 μL
10 × T4 DNA ligase Buffer	2 μL
ddH2O	Up to 20 μL

Table S5. Primers and their sequences used for qRT-PCR in this study.

**Primer**	**Primer sequence (5′→3′)**
Gltx-f-Q-F	GCACCGAAGCCGAACTGGAA
Gltx-f-Q-R	CGTCATCAATGCGGAAGCGAATC
Gltx-Q-F	TGGTGTGGCAGGGCAGTGAT
Gltx-Q-R	TTAGCGGTATTGGCAATGTGGTCTT
A-Q-F	AAGGAGCCACGGACATCACCAT
A-Q-R	ACAGCCAGTCAAATTCTCGCAGTT
B-Q-F	CATGCCTGGAGTGTACCAACTGTC
B-Q-R	TTCACCGCTTCCGTCGCCTT
C-Q-F	GCCAAGTGCCTATCGGTGTTAATAC
C-Q-R	CGCTAAGATTTCCGCTAGGATTTCC
D-Q-F	CGACCAAGCGAGCCAGTTTACC
D-Q-R	GCGGAAGTCAGAATTAGCCAGTCAA
E-Q-F	ACCTTTGCCCTGCCCTACCA
E-Q-R	TCCACCGTCCAGTCCACACT
F-Q-F	GGGCAAATCCCTACCTCCTTCAAT
F-Q-R	TCCACCTCCGAACCACCACA
glbn-Q-F	TGGAACCACCGCTGTCGATCTA
glbn-Q-R	TTTATCCGTACCGCCAAAGGCATAG
H-Q-F	GGGTGTTGGAGGAAATGTGGCATA
H-Q-R	AAATATGGGCTTGGTCTGGATTGGG

J-Q-F	TTGCTCGTGGTGATTGTCCTACTG
J-Q-R	TGCTGTTCCGTTAATAGTGCTTGGT
L-Q-F	GCGGTTCCATCAGTGCCATGTT
L-Q-R	TGTTCTTGGGTGTGGGCTAGGG
N-Q-F	AATTCGTCGCACTGTAATCAAGGA
N-Q-R	AACCATCCGCTTCCAGGACATC
slr0116-Q-F	GTGGAGTAAGTGCGGCTATTGC
ho1-Q-F	CCATCCCATCCTCAGCCACAT
ho2-Q-F	CTTTACGCCAACATCGGGACAA
cysG-Q-F	TGGCATTGAGGTGGAAGTGGTA
chlM-Q-F	GGCATTGGTCTATGGCAGTGAT
slr0116-Q-R	TGACATTGCTGGGACGGATGAA
ho1-Q-R	TTCACTTCTTGCCGCCAGTTG
ho2-Q-R	GCACAGGGAGTAGGCTGGATTA
cysG-Q-R	AGGTGACGGAGGAACTGTAAGC
chlM-Q-R	GCCTCCTCCGTTGGATAGTGAA
rnpB-F	TTTAGAAAACAGCAACCAGT
rnpB-R	GGCAGGAAAAAGACCAACCT

Table S6. Molecular weights of target proteins overexpressed in the mutant strains of *Synechocystis* sp. PCC 6803 derived from either strain PCC 6803 or *Synechococcus elongatus* PCC 7942.

Mutant strain	Protein	Molecular weight (kDa)	Source
g-An	HemA	48.3	PCC 6803
g-Bn	HemB	37	PCC 6803
g-Cn	HemC	35.7	PCC 6803
g-Dn	HemD	59.5	PCC 6803
g-En	HemE	40	PCC 6803
g-Fn	HemF	39.7	PCC 6803
g-Hn	HemH	44.7	PCC 6803
g-Jn	HemJ	24.8	PCC 6803
g-Ln	HemL	46.7	PCC 6803
g-Nn	HemN	54	PCC 6803
g-glbn	GlbN	14.7	PCC 6803
g-gltxn	Gltx	54.8	PCC 6803
g-Af	HemA	49.2	PCC 7942
g-Bf	HemB	26.4	PCC 7942
g-Cf	HemC	35.6	PCC 7942
g-Df	HemD	29.3	PCC 7942
g-Ef	HemE	40.1	PCC 7942
g-Ff	HemF	37.1	PCC 7942
g-Hf	HemH	45.1	PCC 7942
g-Jf	HemJ	24	PCC 7942
g-Lf	HemL	46.9	PCC 7942
g-gltxf	Gltx	54	PCC 7942
